# Forensic significance of intracardiac expressions of Nrf2 in acute myocardial ischemia

**DOI:** 10.1038/s41598-024-54530-x

**Published:** 2024-02-19

**Authors:** Shion Hiyamizu, Yuko Ishida, Haruki Yasuda, Yumi Kuninaka, Mizuho Nosaka, Akiko Ishigami, Emi Shimada, Akihiko Kimura, Hiroki Yamamoto, Miyu Osako, Wei Zhang, Utako Goto, Ten Kamata, Toshikazu Kondo

**Affiliations:** Department of Forensic Medicine, Wakayama Medical University, 811-1 Kimiidera, Wakayama, 641-8509 Japan

**Keywords:** Forensic pathology, Nrf2, Acute ischemic heart diseases, Postmortem diagnosis, Fibronectin, C5b-9, Diagnostic markers, Diagnostic markers

## Abstract

When exposed to oxidative and electrophilic stress, a protective antioxidant response is initiated by nuclear factor erythroid 2-related factor 2 (Nrf2). However, the extent of its importance in the forensic diagnosis of acute ischemic heart diseases (AIHD), such as myocardial infarction (MI), remains uncertain. On the other hand, immunohistochemical analyses of fibronectin (FN) and the terminal complement complex (C5b-9) prove valuable in identifying myocardial ischemia that precedes necrosis during the postmortem diagnosis of sudden cardiac death (SCD). In this study, we investigated the immunohistochemical levels of Nrf2, FN, and C5b-9 in human cardiac samples to explore their forensic relevance for the identification of acute cardiac ischemia. Heart samples were obtained from 25 AIHD cases and 39 non-AIHD cases as controls. Nrf2 was localized in the nuclei of cardiomyocytes, while FN and C5b-9 were detected in the myocardial cytoplasm. The number of intranuclear Nrf2 positive signals in cardiomyocytes increased in AIHD cases compared to control cases. Additionally, the grading of positive portions of cardiac FN and C5b-9 in the myocardium was also significantly enhanced in AIHD, compared to controls. Collectively, these results indicate that the immunohistochemical investigation of Nrf2 combined with FN, and/or C5b-9 holds the potential for identifying early-stage myocardial ischemic lesions in cases of SCD.

## Introduction

The primary objective of forensic pathologists is to determine the precise cause of death. This becomes particularly challenging in instances of sudden death (SD), despite the availability of scientific community’s disseminated professional guidelines, book chapters, and numerous scientific publications addressing this matter. In the event of an SD, a forensic pathologists must perform various procedures to establish the precise cause of death. Sessa et al. presented a flowchart specifically designed for autopsy investigations of sudden cardiac deaths (SCD) in order to support medico-legal investigations^1^.

SCD poses a significant risk to an individual well-being and liveslife, resulting in over 7,000,000 global fatalities annually^2^. Among the causes of SCD, acute ischemic heart disease (AIHD), such as myocardial infarction (MI), is a common cause^3^. Diagnosing SCD due to early myocardial ischemia remains a challenge because ischemic damage often occurs within minutes to hours, and typical gross changes in myocardial tissue do not manifest immediately during this time period^4,5^. In very early phase of myocardial ischemia, histological features, including wavy fibers, nuclear changes, eosinophilia in cardiomyocytes, cytoplastic homogenization, and contraction band necrosis may be observed^6^. Nonetheless, relying solely on postmortem morphological analysis for the diagnosis of early myocardial ischemia-induced SCD lacks specificity, because similar morphological changes can be observed in other diseases and conditions, such as electrocution, amphetamine abuse, and defibrillation^7^.

In standard pathology practice, the diagnosis of AIHD is determined by the presence of two markers: fibronectin (FN) and the terminal complement complex C5b-9^8^. Interpreting FN levels can be challenging because of the influence of various factors such as resuscitation and overall hypoxia. Further, despite demonstrating high specificity, C5b-9 may not account for all instances of suspected AIHD^9–11^. Furthermore, FN and C5b-9 turn positive one and two hours after the onset of ischemia, respectively^9,12,13^. Therefore, accurately diagnosing AIHD within the initial 2-h period remains a challenge. Consequently, ongoing research efforts are focused on enhancing the sensitivity and specificity of pathological AIHD diagnosis by investigating additional markers^14^.

Oxidative stress triggers the activation of the transcription factor nuclear factor erythroid 2-related factor 2 (Nrf2), which plays a vital role in the production of endogenous antioxidant enzymes^15^. Nrf2 is extensively present in several oxygen-consuming organs, such as muscles, the heart, blood vessels, liver, kidneys, and the brain^16^. Nrf2 is a crucial regulator of the antioxidant responses. Upon stimulation by reactive oxygen species (ROS), Nrf2 undergoes molecular changes and translocates from the cytoplasm to the nucleus. The translocated Nrf2 facilitates the transcription of downstream genes including heme oxygenase-1 (HO-1), solute carrier family 7 member 11 (SLC7A11), and glutathione peroxidase 4 (GPX4), whose protein products play significant roles in antioxidant defense and antiferroptosis^17,18^. Nrf2 plays a broad role in reducing oxidative stress, enhancing endoplasmic reticulum stress, repairing impaired mitochondria, regulating inflammation and autophagy, and exhibitinfg cardioprotective effects^19–24^.

The diagnosis of AIHD is a common challenge in routine forensic work. We have previously reported that intracardiac HO-1 expression is a valuable marker for identifying AIHD as the cause of death^25^. However, no reliable markers exist for the diagnosis of AIHD^26^. Therefore, at least a combination of different markers is recommended for the AIHD diagnosis. With the identification of new markers, additional morphological tools may become available for the diagnosis of AIHD. Therefore, in this study, we examined the expression of intracardiac Nrf2 protein in forensic autopsy specimens. Further, we compared the results with the expression of existing markers, FN and C5b-9, to determine whether it could serve as an indicator for the postmortem diagnosis of AIHD.

## Results

### Enhanced intracardiac Nrf2 expression in AIHD group

**Figure 1:**
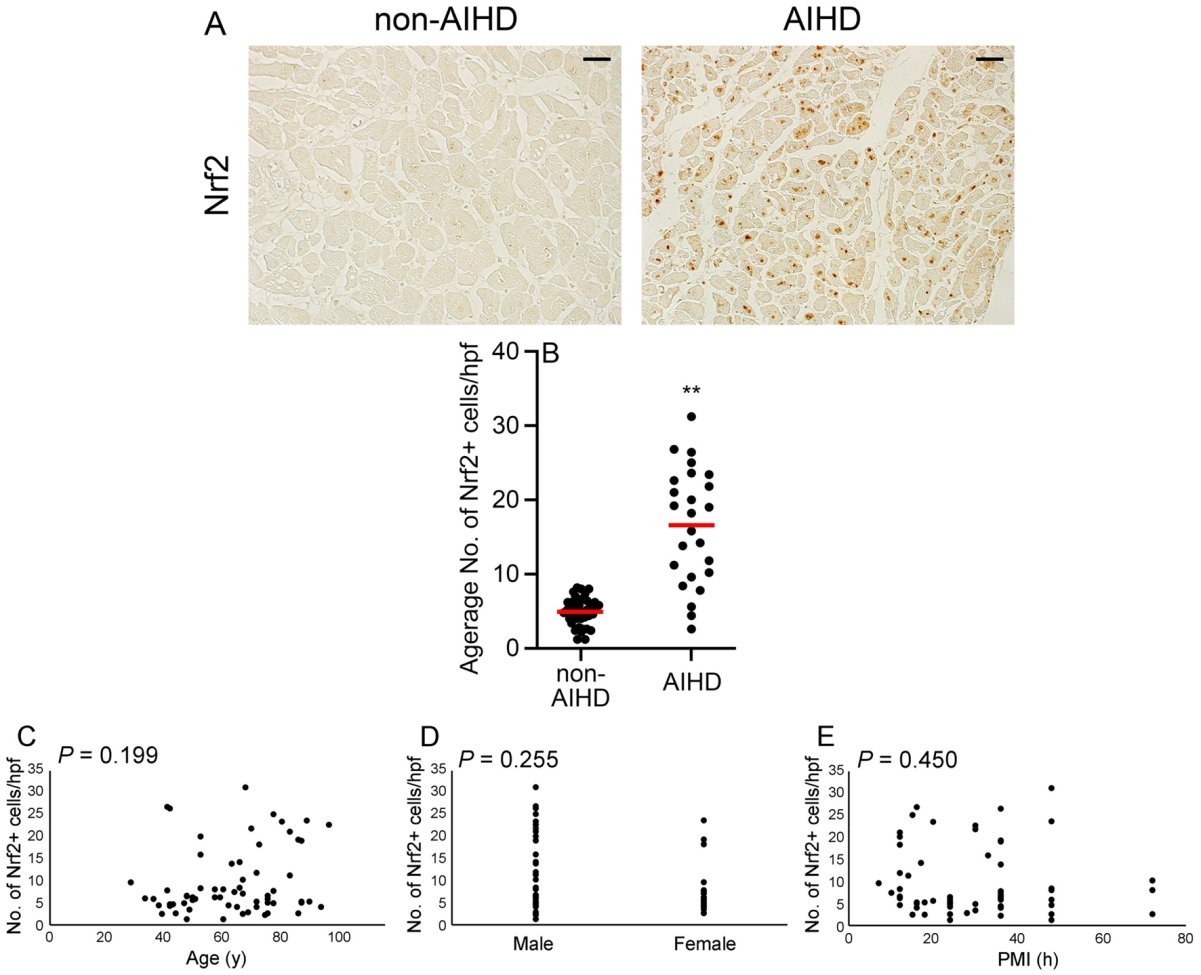
Expression of Nrf2 in the human heart. (**A**) Immunohistochemical analysis using anti-Nrf2. Representative results from hearts of non-AIHD and AIHD groups are shown. Scale bar, 50μm. (**B**) Morphometrical analysis was performed to measure the number of Nrf2^+^ cells in each group. Mean and standard error of the number of Nrf2^+^ cells per high power field (hpf) in each group. **P < 0.01 indicates a statistically significant difference. (**C**) Relationship between age, sex, or PMI and appearance of Nrf2^+^ cells in all cases; *P* values were determined using Spearman's correction coefficient by rank test as previously described^24^.

Immunohistochemical analysis revealed a more intense and diffuses expression of Nrf2 in the nuclei of cardiomyocytes within the AIHD group than in the non-AIHD group (Fig. 1A). Comparison of the AIHD and non-AIHD groups showed significant differences in the number of Nrf2^+^ cells (Fig. 1B). No significant differences were found in age, sex, or postmortem interval (PMI) concerning Nrf2 protein expressions (Fig. 1, C-E).

### Appearance of FN^+^ region in human hearts

**Figure 2:**
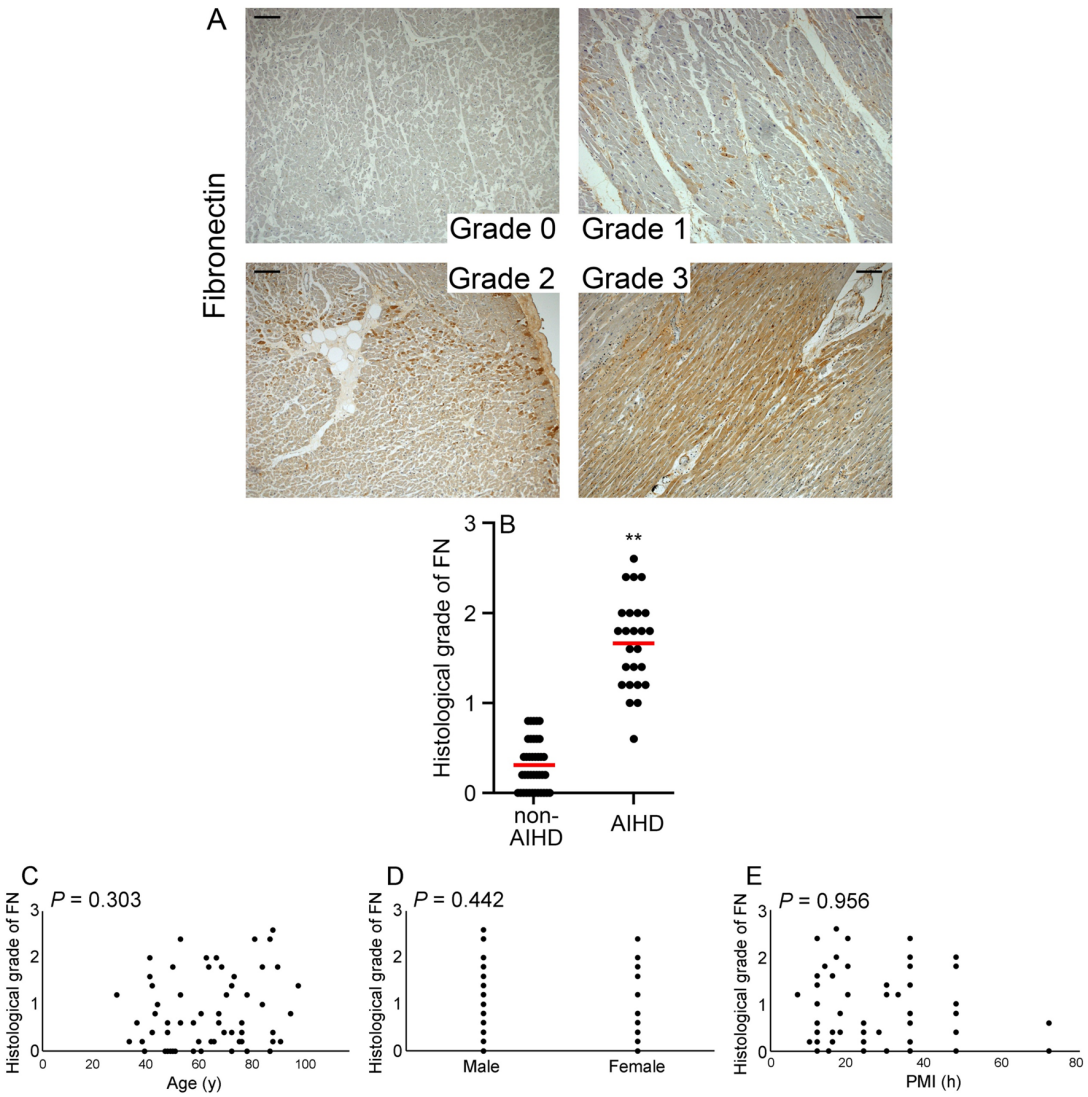
FN expression in human heart. (**A**) Immunohistochemical analysis using anti-FN. Representative results for grade 0 to 3 hearts are shown. Scale bar, 100μm. (**B**) Morphometrical analysis was used to determine the grade of FN^+^ regions in each group. The mean and standard error of the grade of FN^+^ regions in each group are shown. **P < 0.01 indicates a statistically significant difference. (**C**) Relationship between age, sex, or PMI and the occurrence of FN^+^ regions in all cases; *P* values were determined using Spearman's correction coefficient by rank test as previously described^24^.

A comparison of the grade of FN^+^ regions in the AIHD and non-AIHD groups showed that the grade in the AIHD group was significantly higher than that in the non-AIHD group (Fig. 2A and B). No significant differences in age, sex, or PMI were identified with respect to FN expression (Fig. 2C–E).

### Appearance of C5b-9^+^ regions in human hearts

**Figure 3:**
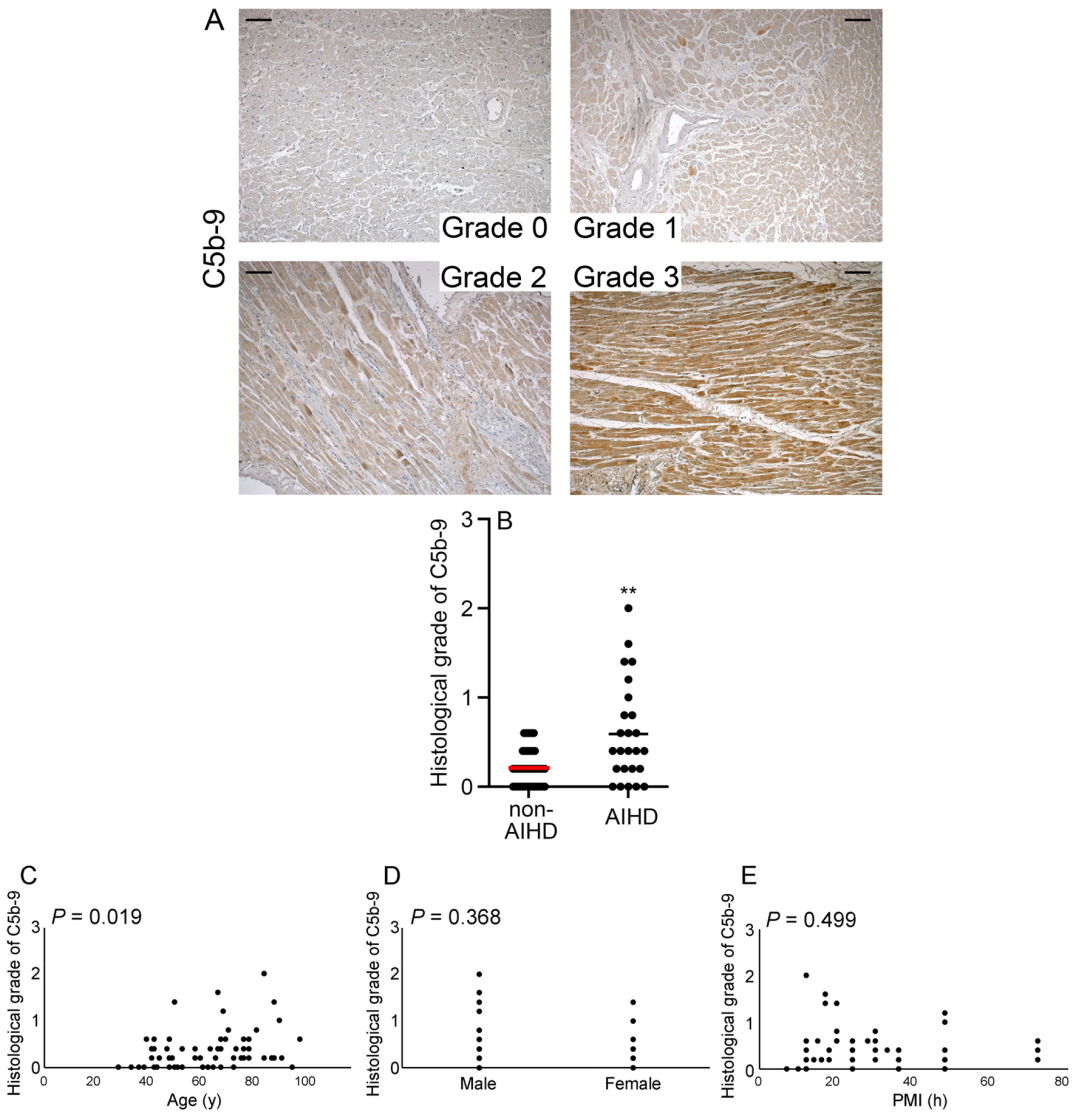
C5b-9 expression in human heart. (**A**) Immunohistochemical analysis using anti- C5b-9. Representative results for grade 0 to 3 hearts are shown. Scale bar, 100μm. (**B**) Morphometrical analysis was used to determine the grade of C5b-9^+^ regions in each group. The mean and standard error of the grade of C5b-9^+^ regions in each group are shown. **P < 0.01 indicates a statistically significant difference. (**C**) Relationship between age, sex, or PMI and the occurrence of C5b-9^+^ regions in all cases; *P* values were determined using Spearman’s correction coefficient by rank test as previously described^24^.

A comparison of the grades of C5b-9^+^ regions in the AIHD and non-AIHD groups demonstrated that the grade in the AIHD group was significantly higher than that in the non-AIHD group (Fig. 3A and B). However, very few regions of the specimens in the AIHD group exhibited the highest grade of 3. Additionally, our analysis revealed significant age-related differences in C5b-9 protein expression (Fig. 3C, P = 0.019). However, no significant differences were found in sex and PMI concernig C5b-9 protein expressions (Fig. 3D and E).

## Discussion

The majority of acute myocardial ischemia often result in lethal ventricular arrhythmias, and the short time between ischemic attack and death may be unrelated to specific structural abnormalities of the heart (macroscopic and microscopic) that can be assessed by common methods. Thus, reliably attributing the cause of death to acute myocardial ischemia is one of the most significant challenges for forensic pathologists^27^.

Recent studies on the postmortem diagnosis of myocardial ischemia have immunohistochemically examined markers that either accumulate (such as fibronectin) or leak (such as troponin, myoglobin, and S100A1) into human myocytes during ischemic injury^10,14,28,29^. The rationale behind conducting immunohistochemical examinations is that ischemia can cause damage cell membranes, contractile proteins, cytoskeleton, and intracellular organelles within a relatively short timeframe. Consequently, antibodies targeting different cardiomyocytes components could be utilized. Nonetheless, the time required to detect the depletion and accumulation of these changes in cardiomyocytes remains excessively long. Despite the use of a combination of different markers, waiting at least for one hour is necessary after the onset of symptoms and death to observe histochemical alterations. The diagnostic potential of markers/mediators related to early inflammation, such as CD15, Il-1ß, IL-6, TNF-α, IL-15, IL-8, MCP-, ICAM-1, CD18, and tryptase, in the context of MI, has been extensively explored and deliberated upon^30–33^.

Multiple markers, rather than a single marker, have been demonstrated to be useful in detecting myocardial ischemia^14^. Immunohistochemistry has been used to investigate various constituent proteins of cardiomyocytes, such as the cytoskeleton^34^, contractile proteins cTnI, cTnT^35,36^, and cTnC^26^, other myoglobins^26,37,38^, cardiac fatty acid binding protein (H-FABP)^26^, and desmin^34^. Fibronectin^10,13,14,28^, C5b-9^26,36–41^ or S100A1 protein^29^ leak after ischemic injury. A significant number of these investigations have been conducted in human subjects (autopsy cases), and the general consensus from these studies is that no single marker exhibits sufficient sensitivity and specificity for the early detection of myocardial ischemia. Additionally, challenges and uncertainties arise when attempting to classify a positive range of individual proteins.

The membrane attack complex, C5b-9, is the most extensively examined marker for the forensic diagnosis of early MI. The C5b-9 complex represents the final outcome of complement system activation. They interact with and harms cell membranes, leading to direct cell lysis^42^. The first study of the C5b-9 complex determined that it was specific for necrosis, as this marker was immunohistochemically detected at the MI site^41,43^. This antigen is detected in cases without histological signs and/or in myocardial ischemic tissue 30–40 min after initiation of ischemic injury^12,37,44^. The findings of this experiment suggest that pathologists concur with the potential usefulness of this approach in the early detection of ischemic myocardial injury. However, in addition to the uncertainty regarding the grading of C5b-9 positive lesions, a significant relationship between age and C5b-9 expression was observed under the conditions of the current analysis.

Importantly, the Nrf2 signaling pathway has been linked to safeguarding against myocardial injury induced by ischemia/reperfusion (I/R), protecting against oxidative stress, and addressing electrolytes imbalances^45^. Studies has demonstrated that increasing the Nrf2 levels in astrocytes inhibits apoptosis^46^ and that Nrf2 translocates into the nucleus to regulate gene expression, crucial for oxidative stress and cardiac apoptosis induced by hypoxia/reperfusion (H/R)^47–49^. Traditionally, Nrf2 has been recognized as a fundamental controller responsible for preserving the balance of redox reactions and participating in the transcriptional activation of downstream antioxidant enzymes^15^. HO-1, an antioxidant enzyme induced by Nrf2, protects cells from oxidation-induced injury^50^. Studies have indicated that excessive expression of HO-1 protects the heart against oxidative stress and apoptosis induced by I/R^51^. In the presence of oxidative stress, Nrf2 triggers the expression of HO-1, shieldings cells from oxidative stress thorough heme breakdown. HO-1 has been previously suggested as a valuable marker for the forensic diagnosis of acute myocardial ischemia^25^.

Although no perfect immunohistochemical staining method exist for diagnosing AIHD, a combination of multiple markers can improve the ability of forensic pathologists to identify ischemic areas in the absence of macroscopic or microscopic evidence of necrosis. Nevertheless, the analysis of data obtained for each individual marker cannot be isolated from the comprehensive assessment of factors that might impact the expression of each marker, such as cardiopulmonary resuscitation and agonal artifacts. Therefore, utilization a combination of diverse markers, including Nrf2, HO-1, FN, and C5b-9, will facilitate a more objective and precise evaluation of early myocardial injury. This approach contributes to advancements in the postmortem diagnosis of AIHD when macroscopic and microscopic evidence is inadequate.

Moreover, magnetic resonance imaging (MRI) has long been recognized as an advanced imaging technique with specific applications and is valued as a potent diagnostic tool; however, studies regarding its application to forensic practices^52–54^ are limited. Given its non-invasive nature, post-mortem MRI (PM-MRI) has emerged as a promising technique for investigating and highlighting findings that may not be discernible through routine autopsy procedures^55^. However, the interpretation of PM-MRI, especially in cases involving contrast agent use, demands sophisticated expertise. This is particularly pertinent when substantiating cardiovascular-related deaths in a forensic context. Furthermore, numerous experimental studies have elucidated the roles of various microRNAs (miRNAs) and small noncoding RNAs in regulatory processes at the cardiac level, playing a pivotal role in cardiovascular disease (CVD)^56–59^. Despite the acknowledged potency of miRNAs as gene regulators, their underlying mechanisms remain unclear. Two studies were conducted using post-mortem samples, underscoring the significance of research in this area, given the advantages of utilizing autopsy tissues^60,61^. For instance, in an experimental study focused on monitoring heart tissue after MI, it was feasible to precisely select the area affected by the infarction. Additionally, the analysis of formalin-fixed paraffin-embedded (FFPE) tissues provides the opportunity to utilize post-mortem samples collected for other purposes, such as histological investigation, to determine organ damage. Therefore, forensic institutions are invaluable sources of samples.

## Limitations of the study

This study has some limitations. We did not account for potential influences from factors, such as medication regimen and BMI in each case, which might affect the data for both groups. However, in all cases, in addition to sex, and time since death, the cause of death was carefully diagnosed based on a complete autopsy, histology, toxicology, and diatom examination. This highlights the difficulty of conducting tests on human subjects, which is inherently less uniform compared to animal studies. However, the present study provides sufficient evidence supporting the forensic applications of Nrf2 expression investigations.

## Methods

### Antibodies (Abs)

In this study, following polyclonal or monoclonal Abs (pAbs or mAbs) were used for immunohistochemical analyses in the present study: rabbit anti-human Nrf2 pAbs (bs-1074R, Bioss antibodies, Woburn, MA), rabbit anti-human fibronectin pAbs (15613-1-AP, Proteintec, Rosemont, IL), rabbit anti-human C5b-9 pAbs (bs-2673R, Bioss antibodies).

### Human heart tissue samples

A total of 64 human forensic autopsy cases were included in this study (42 males; 22 females) with a PMI of less than 72 h, chosen based on autopsy documents. The individual ages ranged from 29 to 100 years (mean age, 65.2 ± 17.8), and the mean age of males and that of females was 64.7 ± 15.7 and 66.2 ± 21.7 years, respectively. In each case, the cause of death was carefully determined through a comprehensive examination, including complete autopsy, histology, toxicology, and diatom tests. Cases were divided into two groups: 25 AIHD cases with advanced sclerosis and/or stenosis in the coronary artery and 39 others, including drowning (18 cases), fire death (6 cases), hanging (3 cases), traumatic shock (3 cases), asphyxia (2 cases), severe head injury (2 cases), and singular case of acute drug intoxication, acute carbon monoxide intoxication, pneumonia, peritonitis, and nicotine addiction. The detailed profiles of all cases (sex, age, and PMI) are shown in Table 1. We selected the following study groups: AIHD, 25 cases; non-AIHD, 39 cases. The AIHD group comprised sudden cardiac death with gross and/or histopathological signs of myocardial infarction. In all cases of AIHD, the cause of death was acute myocardial infarction, Inclusion criteria for this group were histopathological signs (HE staining) of early ischemia, such as contraction band necrosis, cardiomyocyte coagulation necrosis, polymorphonuclear leukocyte infiltration, and/or wavy fibers. Cases showing putrefaction, carbonization, and traumatic injury of the heart, and cases after percutaneous coronary revascularization procedures and/or coronary artery bypass grafting were excluded. The control group (non-AIHD group), included 39 cases with no clinical history or macroscopic and microscopic findings of cardiac pathology from various causes of deaths described in Table 1.

**Table 1:** Forensic autopsy cases.

Cause of death	n	Male/Female	Age (years)		PMI (hours)	
			Range	Mean ± SD	Range	Mean ± SD
AIHD	25	19/6	29–100	67.6 ± 18.7	7–72	28.6 ± 16.0
Non-AIHD	39	23/16	34–97	63.7 ± 17.3	10–72	29.4 ± 15.0
Drowning	18					
Fire death	6					
Hanging	3					
Traumatic shock	3					
Asphyxia	2					
Severe head injury	2					
Drug and poisoning	2					
Acute CO intoxication	1					
Pneumonia	1					
Peritonitis	1					
Total	64	42/22	29–100	65.2 ± 17.8	7–72	29.1 ± 15.3

### Histopathological analyses

Heart samples were fixed in 4% formaldehyde buffered with PBS and embedded in paraffin. Immunohistochemical analysis were carried out using anti-Nrf2, -FN, or -C5b-9 Abs. Deparaffinized sections were immersed in 0.3% H_2_O_2_ in methanol for 30 min to eliminate endogenous peroxidase activity. The sections were further incubated with PBS containing 1% normal serum corresponding to the secondary Abs and 1% BSA to reduce nonspecific reactions. Subsequently, the sections were incubated with primary antibodies at 4 °C overnight. After incubation with biotinylated secondary antibodies, the immune complexes were visualized using the Catalyzed Signal Amplification System (Dako Cytomation, Kyoto, Japan), according to the manufacturer’s instructions. As a negative control, sections were incubated with normal rabbit IgG instead of primary antibodies and no positive signal was detected, indicating the specificity of the antibodies.

### Morphometrical analysis

Morphometry was performed for semiquantitative evaluation, as previously reported^62,63^. For Nrf2, five high-power microscopic fields (× 200, 0.6 × 0.6 mm each) were randomly chosen in each section. The number of Nrf2^+^ cells was counted, and the average number was evaluated in each heart specimen. Positive reactions to FN and C5b-9 were semiquantitatively classified into: group 0, negative reaction; group 1, light reaction; group 2, moderate reaction; and group 3, strong reaction.

### Statistical analysis

The AIHD and control groups were compared using a two-sided unpaired Student’s *t* test to identify significant differences (*P* < 0.05 considered significant). All statistical analyses were performed using Statcel3 software under the supervision of a medical statistician as previously described^25^.

### Ethical approval

This study was approved by the Research Ethics Committee of Wakayama Medical University (No. 3176). All the procedures were performed in accordance with the principles of the Declaration of Helsinki. Moreover, this study was conducted using past autopsy records, and informed consent was not obtained from bereaved family members for the use of these records. Therefore, we conducted this study in accordance with the "Ethical Guidelines for Medical Research Involving Human Subjects (enacted by the Ministry of Health, Labor, and Welfare in Japan), section 12–1 (2) (a) (c).” This was a de-identified study using archived tissue obtained from judicial autopsy cases, and information on the implementation of the study was posted on our website (https://www.wakayama-med.ac.jp/dept/igakubu/160420/index.html). If they were requested to refuse the use of the samples for research, they were excluded from the study. In addition, the review board of the Research Ethics Committee of Wakayama Medical University waived the need for written informed consent from relatives of the individuals studied in accordance with national legislation and institutional requirements.

## Data Availability

The authors declare that all data are available in the article file, or available from the corresponding author (Toshikazu Kondo) upon reasonable request.
